# Modern Sociology

**Published:** 1900-11-24

**Authors:** 


### Modern Sociology.

### SOME DANGEROUS TRADES.

### II.—Inflammable Paints.

"When ships come into dock or alongside a wharf or quay for repair the chief anxiety of the owner or the master is to get away again. That 'time is money' is true of ships probably more than of any profit-making machine in existence. To meet this demand of speed in the repairing of ships much ingenuity has been expended and many devices invented. Among them the invention of quick-drying paints is one of the most ingenious, if not one of the most injurious."

These are the opening words of the report on the use of inflammable paints. Paint is necessary, not for ornament, but to save wood and iron from the wearing effects of weather. Ordinary paints are composed of colouring material mixed with oil, while the new and inflammable paints are composed of the colouring material *plus* a composition containing methylated spirit, petroleum spirit, or benzine. They are very volatile, very inflammable, the flash-point of some being under 45° Fahr. When it is remembered that the present flash-point of petroleum —73°—is considered by many to be too low, the danger of working with these paints can be inferred. The advantages are obvious. A ship painted with spirit paints will be ready to leave the dry dock in three days, when if painted with the old-fashioned paint the drying process would keep her there for a week. "A foreman painter on the Tyne informed the Committee that he had known a ship to be painted in dry dock in one day." But there are two great disadvantages—the danger of the outbreak of apparently spontaneous fire and the risk of intoxication from the inhalation of the fumes of the spirit. This is sometimes followed by unconsciousness and asphyxiation.

The more modified symptoms of the latter danger are often seen, such as a temporary loss of the faculties, and other effects which simulate those of ordinary drunkenness. When inhaled in considerable quantities the vapour produces nausea and vomiting, loss of appetite, colic, extreme torpor, feebleness of the muscles, bleeding at the nose and ears, and temporary dementia.

The existence of the one danger increases the other, that of fire. An outbreak of fire is much more likely to occur where the workers are more or less stupefied by inhaling an intoxicating vapour, and when, through the unconsciousness of the intoxicated man, a fire occurs, the consequences are likely to be serious. Add to this that these paints are particularly handy for painting the confined parts of a ship, where it is difficult to get a thorough draught to dry the paint, and in which, since they are not lighted, the worker requires artificial light, and it may be guessed how frequently accidents are liable to occur. Sometimes when men are engaged in painting the outside of a ship with these inflammable paints, the ship's side suddenly, for no apparent reason, often bursts into flames. The flames rise rapidly, giving off large volumes of smoke and great heat, and as suddenly as they arose they go out. It is usually found out on investigation that there has been a naked light too near the freshly-painted ship's side, and that the volatile spirit, evaporating from the paint, has caught the fire, and communicated it to the rest of the paint. When conflagrations take place in the open air, they are often unaccompanied by any casualty; but the case is very different when the fire arises in a confined space. One foreman painter expressed the opinion that the hold of a ship was too confined a space to be treated safely with these paints. Yet the hold is usually a fairly large space. The smallest is big enough to permit of three or four men being at work at the same time, which is of itself a safeguard against serious accident; and it is generally possible for the men to stand upright, and to work by daylight. How much more dangerous must it be, then, to use these paints in small spaces where a man must go alone, must work stooping or lying down, must creep in and out of manholes to go to his work, and must use artificial light? "Horrible cases of stupefaction, in the first instance," says the Report, "and fire subsequently followed by months of illness from burning, or ultimate death, have been brought to the notice of the Committee."

These inflammable or spirit paints are more expensive than ordinary paints, but, by way of compensation, less of them is required. They are about twice as dear, but one coat of them will serve the same purpose as two coats of the ordinary oil paint. At first the men objected to using these paints, and demanded pay at the rate of "time and a quarter" or "time and a half" when they did so; but from the report of the Committee it does not appear whether this was due to the danger attaching to their use or to a feeling that work which brought about the same result as the old method, even if less laborious, should have the same pay or nearly so. In any case, this objection threatened to do away with the use of the spirit paints. To meet this the patentees, it is said, sent their representatives to give the men "drop allowances," or, in other words, bribes to the men to persuade them to continue their use. "It appears to the Committee that if so much pressure be required to establish this commodity in the market, its intrinsic merits cannot be so great as to warrant its universal introduction into the ship-repairing industry."

The Committee confine themselves, however, to recommending that no confined space on board ship, nor any place to and from which the workers must pass through a manhole, should be painted with spirit composition or with any paint the flashing point of which is below 100° Fahr. when tested by Abel's apparatus. No such paint should be used by a workman carrying a naked light, nor upon a surface between which and a naked light there is no protective medium. They advise that no one should be allowed to work with these paints for more than five hours a day, nor for longer than two and a half hours at a time with a break of one hour between times. Even with this limit temporary cessations from work would still be necessary, as men often feel faint and giddy and have to go into the open air until the symptoms pass off. Where less than three men are employed in the work the Committee think that they should be visited by a foreman or other responsible person at intervals of not more than one hour. A difficulty at present exists in that all the places where ships are repaired are not under the Factories Acts. Ships in shipbuilding yards are subject to the Factory and Workshops Act, but ships in docks or lying at quays or wharves are subject to them only with regard to loading and unloading; while ships at anchor, in rivers, or harbours are not under the Factory Acts at all. The Committee, therefore, recommend that all ships while undergoing repair should be looked on as factories.

